# Out of the Blue: Methemoglobinemia Associated With the Use of Amyl Nitrite in Rush Poppers for Erectile Stimulation

**DOI:** 10.7759/cureus.41201

**Published:** 2023-06-30

**Authors:** Iyad Y Idries, Vasilii Khristoforov, Sushant Duddala, Y V Duong, Mohammad Zaman

**Affiliations:** 1 Internal Medicine, Brookdale University Hospital Medical Center, Brooklyn, USA; 2 Research, Prathima Institute of Medical Sciences, Nagunur, IND; 3 Research, University of Debrecen Medical School, Debrecen, HUN; 4 Critical Care Medicine, Brookdale University Hospital Medical Center, Brooklyn, USA

**Keywords:** poison control center, oxidation of hemoglobin, amyl nitrite, methylene blue, chocolate-colored blood, foaming at the mouth, erectile stimulation, rush, nitrite-containing poppers, methemoglobinemia

## Abstract

We report a clinical scenario involving a 51-year-old male patient with a history of prediabetes and gastritis who exhibited altered mental status following the consumption of poppers, a supplement containing nitrites, which is used for erectile stimulation. Shortly after the ingestion, the patient experienced convulsions, foaming at the mouth, and subsequently developed altered mental status and severe respiratory distress. The diagnosis of methemoglobinemia was confirmed based on elevated methemoglobin levels on venous blood gas analysis. Notably, the patient's blood had a chocolate-colored appearance upon admission, which is a characteristic finding in methemoglobinemia. Prompt recognition and management, including the administration of methylene blue, led to the resolution of symptoms. This case highlights the potential complications associated with the consumption of poppers and emphasizes the importance of early intervention.

## Introduction

Amyl nitrites, often referred to as "poppers", are recreational drugs commonly used for their vasodilatory properties and ability to stimulate erectile function [1]. A rare yet potentially severe complication associated with the use of poppers is methemoglobinemia [2,3]. This report discusses a case of a patient diagnosed with methemoglobinemia after the ingestion of poppers, emphasizing the need to consider this condition in patients exhibiting altered mental status and respiratory distress after the use of poppers [4].

## Case presentation

A 51-year-old male with a history of prediabetes and gastritis presented to the emergency department with altered mental status and severe respiratory distress. According to his partner, the patient had consumed one bottle of a nitrite-containing supplement called Rush, commonly known as poppers, by oral ingestion in a hotel bathroom. Shortly after the ingestion, he experienced convulsions, foaming at the mouth, and became cyanotic. His blood was noted to be chocolate-colored on admission, consistent with methemoglobinemia.

On examination, the patient was severely short of breath, and his oxygen saturation was 77% despite receiving oxygen therapy via a bag valve mask. His blood pressure was 86/55 mmHg, temperature was 35.9 °C, and respiratory rate was 30 breaths per minute. Physical examination revealed marked cyanosis.

Laboratory investigations, including a venous blood gas analysis, showed methemoglobinemia levels above 30% of the normal level, exceeding the test's maximum detection limit. His pO_2_ was 36.1 mmHg, pH was 7.33, PCO_2_ was 40.0, and carboxyhemoglobin levels were normal (<0.1%). The patient's O_2_ hemoglobin saturation was at 77.0% on a bag valve mask. These findings confirmed the diagnosis of methemoglobinemia.

## Discussion

**Figure 1:**
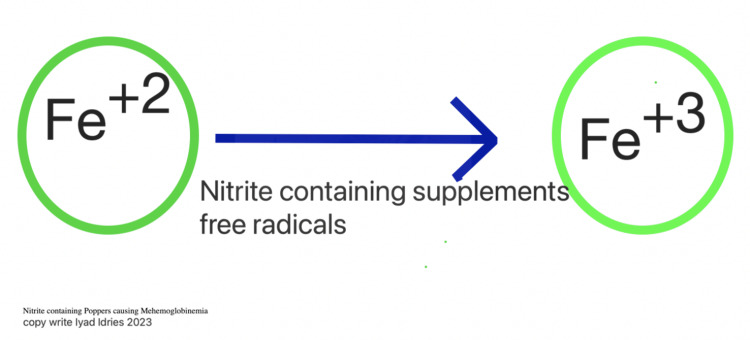
Effects of nitrite-containing poppers on hemoglobin

Methemoglobinemia is a condition resulting from the oxidation of hemoglobin, which converts the iron molecule from the ferrous state (Fe^+2^) to the ferric state (Fe^+3^) [5,6], as illustrated in Figure 1. This conversion reduces the oxygen-carrying capacity of the blood. Alkyl nitrites, commonly known as poppers, can induce methemoglobinemia by prompting this oxidation process [6,7], which leads to the elevation of methemoglobin levels on arterial blood gas [8]. Other factors that cause the process of oxidation include exposure to vegetables and groundwater that have been exposed to nitrogenous fertilizers [7], Drug-induced methemoglobinemia secondary to medications such as primaquine, dapsone, and benzocaine has also been described [9]. This conversion to methemoglobin makes it lose its affinity to oxygen, leading to hypoxia of the tissue and severe cyanosis [10,11]. In our patient, signs such as altered mental status, severe respiratory distress, cyanosis, and distinctive chocolate-colored blood supported the diagnosis of methemoglobinemia.

**Figure 2:**
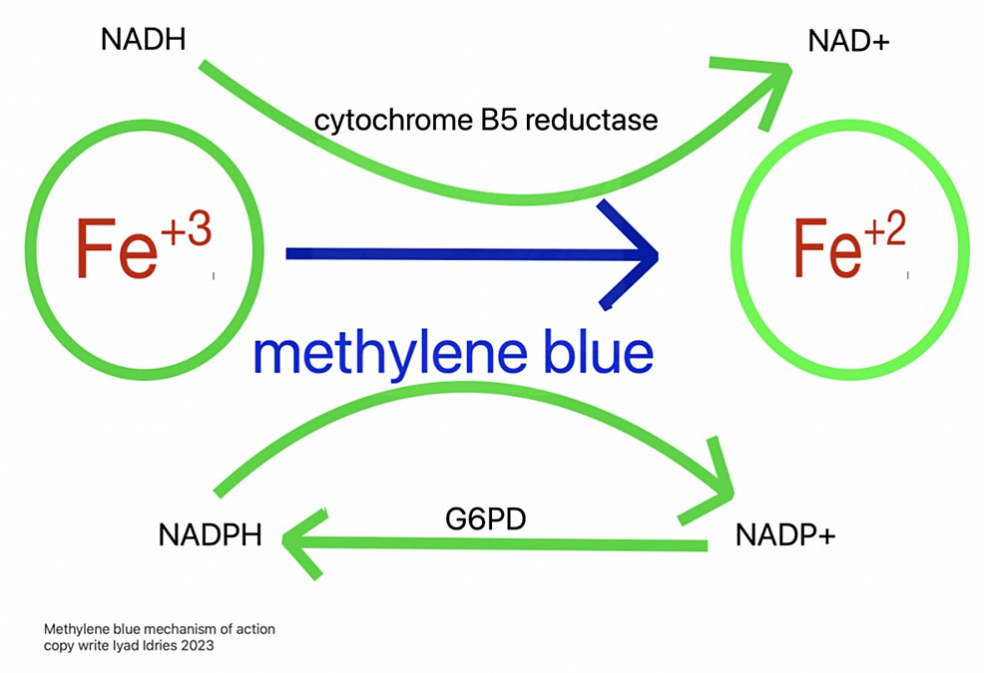
Mechanism of action of methylene blue

Upon admission to the ICU, the patient had to be intubated due to hypoxic respiratory failure, even though his oxygen saturation lingered in the 80s. The Poison Control Center advised administering 100 mg of methylene blue, which reverses methemoglobinemia by reducing ferric iron (Fe^+3^) back to its ferrous state (Fe^+2^), thereby increasing its affinity for oxygen [7,12], as depicted in Figure 2. The patient subsequently developed hypertension due to the effect of methylene blue to inhibit guanylate cyclase, thereby decreasing C-GMP and vascular smooth muscle relaxation [13], likely a side effect of methylene blue [2,12], and a transient blue discoloration of urine. To manage the hypertension, a propofol drip was initiated and an intravenous push of enalaprilat was administered. The patient also presented with acute kidney injury (AKI), likely a result of the ingested toxin [14].^ ^The treatment plan included intravenous fluid therapy with lactated Ringer's solution at a rate of 125 cc/hr, coupled with close monitoring of urine output. Various imaging studies, including a CT scan and CTA of the head and neck, were conducted and ruled out stroke, while an MRI performed during the admission was negative for acute changes. A seizure disorder was suspected, prompting the initiation of Keppra therapy, but was later discontinued after an EEG showed no abnormalities.

During the patient's hospitalization, new-onset hypertension was observed, likely due to the methylene blue administration [4,6].^ ^This was successfully managed initially with propofol and then with enalaprilat followed by amlodipine 10 mg.

## Conclusions

This case report highlights the potential complications of the use of poppers and the consequent development of methemoglobinemia. Rapid identification of the condition - marked by altered mental status, severe respiratory distress, cyanosis, and chocolate-colored blood - is vital for commencing proper treatment. Methylene blue administration effectively reversed methemoglobinemia and ameliorated the symptoms; alternatively, ascorbic acid can be used as an adjuvant if methylene blue is contraindicated. Medical professionals must stay alert to recognize and manage methemoglobinemia, particularly in patients with pre-existing conditions. Public awareness campaigns and educational initiatives regarding the risks of poppers can help foster safer sexual practices and minimize adverse effects.

Further research is required to deepen our understanding of the pathophysiology, most effective diagnostic methods, and optimal treatment strategies for methemoglobinemia resulting from the consumption of poppers and other substances including vegetables and groundwater, as methylene blue can lead to side effects including severe hypertension, especially after repeated administration.
